# Machine Learning’s Application in Deep Brain Stimulation for Parkinson’s Disease: A Review

**DOI:** 10.3390/brainsci10110809

**Published:** 2020-11-01

**Authors:** Jeremy Watts, Anahita Khojandi, Oleg Shylo, Ritesh A. Ramdhani

**Affiliations:** 1 Department of Industrial and Systems Engineering, University of Tennessee, Knoxville, TN 37996, USA; gkm819@vols.utk.edu (J.W.); khojandi@utk.edu (A.K.); oshylo@utk.edu (O.S.); 2 Department of Neurology, Donald and Barbara Zucker School of Medicine at Hofstra/Northwell, Hempstead, NY 11549, USA

**Keywords:** machine learning, deep brain stimulation, Parkinson’s disease

## Abstract

Deep brain stimulation (DBS) is a surgical treatment for advanced Parkinson’s disease (PD) that has undergone technological evolution that parallels an expansion in clinical phenotyping, neurophysiology, and neuroimaging of the disease state. Machine learning (ML) has been successfully used in a wide range of healthcare problems, including DBS. As computational power increases and more data become available, the application of ML in DBS is expected to grow. We review the literature of ML in DBS and discuss future opportunities for such applications. Specifically, we perform a comprehensive review of the literature from PubMed, the Institute for Scientific Information’s Web of Science, Cochrane Database of Systematic Reviews, and Institute of Electrical and Electronics Engineers’ (IEEE) Xplore Digital Library for ML applications in DBS. These studies are broadly placed in the following categories: (1) DBS candidate selection; (2) programming optimization; (3) surgical targeting; and (4) insights into DBS mechanisms. For each category, we provide and contextualize the current body of research and discuss potential future directions for the application of ML in DBS.

## 1. Introduction

Deep brain stimulation (DBS) has evolved into an effective therapeutic agent for Parkinson’s disease (PD) patients suffering from medication refractory tremor, motor fluctuations, and/or troublesome dyskinesia. The stimulation is delivered through electrodes that are stereotactically implanted into either the subthalamic nucleus (STN) or the globus pallidus internus (GPi). Targeting is achieved with intraoperative image guidance (magnetic resonance imaging (MRI) or computed tomography (CT)) [1,2,3]. Neurophysiological mapping via microelectrode recording (MER) is often utilized to improve targeting precision. DBS therapy is engineered by customizing the size, shape, and intensity of the stimulation field with newer electrodes (tripartite-segmented electrodes from Abbott^®^, Medical Infinity^TM^, Austin TX, USA, and Boston Scientific^®^, Vercise^TM^, Marlborough, MA, USA), producing larger therapeutic windows and increasing the combinations of programming options [4,5].

Currently, high frequency continuous stimulation drives therapeutic benefit and requires time-consuming programming sessions, often relying on a trained clinician culling from experience and established programming approaches [6,7] to achieve the best clinical outcomes. However, stimulation-related side effects and suboptimal clinical responses can be limiting factors. Adaptive DBS (aDBS), which is a closed loop stimulation system [8] that uses a physiological biomarker of the parkinsonian state to automatically adjust the stimulation level and location, can provide an alternative to traditional DBS. This offers the possibility of more efficiently treating patients while reducing the challenges inherent in the current programming approaches. Furthermore, the recent advances in DBS technology, including improvements in spatial control from segmented electrodes and image guided programming, along with brain sensing (a likely precursor for closed loop DBS) can benefit from the application of data driven modeling to enhance clinical decision making and outcome prediction. In this review, we examine the applications of machine learning approaches for DBS in PD.

Machine learning (ML) is a subfield of artificial intelligence (AI) that identifies patterns within data sets. The application of ML in healthcare is becoming more widespread to augment the work of clinicians [9]. For instance, ML models have been created to identify anatomical structures through medical imaging [10], and to optimize pervasive sensing in healthcare informatics [11]. In the context of PD, much work has been done to successfully use ML/AI. This includes detecting PD symptoms for diagnosis purposes (e.g., [12]), predicting disease progression and patient response to medication using motor symptoms (e.g., [13]), optimizing clinical assessments through leveraging wearable sensors (e.g., [14]), and providing medication regimen planning (e.g., [15]).

Traditionally, computer algorithms execute explicit, specifically written code to interpret data and make decisions. ML is a computer analysis subfield that distinguishes itself from traditional methods by seeking to “learn” the relationships that underscore data without exact instructions [16,17]. Simply put, ML is an automated method that uncovers patterns in the data and utilizes those patterns to make predictions and/or facilitate decisions. ML has a wide domain of uses but excels in tasks that have large and complex data sets [16]. Generally, ML algorithms are categorized into two subtypes, namely, supervised and unsupervised learning.

In supervised learning, the algorithm’s general goal is to learn a mapping of inputs to outputs. To accomplish this task, the algorithm is provided a training set of input–output pairs. These inputs are generally multi-dimensional vectors representing features of the data. The response variable, also known as a dependent variable, is the output of interest that is associated with these features. As the true values of the response variable, namely the ground truth, have already been identified by a human, supervised learning algorithms frequently are attempting to approximate human performance [18]. The response variable may be discrete (often called a label) or continuous, resulting in a classification or a regression task, respectively [17]. The performance of a supervised learning algorithm is objectively evaluated on a testing set, a subset of the data that is never examined by the algorithm when training. This allows for quantifying the generalizability of the learned model to unseen data. Dependent on the type of task (classification vs. regression), the model performance is described by diagnostical statistic measurements, such as accuracy, F1 score, or the area under the receiver operating characteristic (AUC) for classification, or metrics such as mean squared error or mean absolute error for regression. The descriptions of these metrics are provided in Appendix A. Examples of supervised learning algorithms include random forests (RF) [19], neural networks (NN) [20], support vector machines (SVM) [21], naïve Bayes (NB) [22], and k-nearest neighbor (KNN) [23].

In unsupervised learning, the algorithm’s general goal is to find trends in data given only inputs. Unsupervised learning tends to be a more difficult process, as there are no known ground truths to predict. Examples of unsupervised learning algorithms include clustering [24], density estimation [25], and some neural networks [20]. Each technique requires different methods to determine the model’s performance. For instance, in clustering, data is separated into distinct groups that ideally share characteristics. A clustering algorithm’s performance may then be analyzed by how close its group members are to one another (intra-cluster distance) or how close the members are to another cluster (inter-cluster distance). While benchmarking unsupervised learning algorithms can be challenging, unsupervised learning allows for the analysis of data sets that are completely incompatible with supervised learning algorithms. Unsupervised learning is well suited for situations where ground truths cannot be identified or in which human experts cannot determine trends. Under these situations, unsupervised learning can propose trends in the data that can lead to meaningful insights when interpreted by experts.

The efficacy of DBS in the treatment of PD is well established [26,27,28,29]. Many of the potential benefits of ML in strategic PD treatment planning have been considered [12,13,14]; however, these reviews do not generally explore the role of ML in DBS. Specific surgical or treatment topics relating to ML in DBS have been explored [30,31,32,33]. However, they do not explore all facets of ML in DBS, and frequently only provide brief considerations of its application. As such, identification of the best performing models, as well as gaps in the existing body of literature, could help guide future exploration in using the powerful tool of ML. Here, we review the applications of ML approaches for DBS in PD. We compartmentalize these approaches into four groups: (1) DBS candidate selection; (2) programming approaches; (3) surgical targeting; and (4) insights into DBS mechanisms.

## 2. Materials and Methods

**Figure A1:**
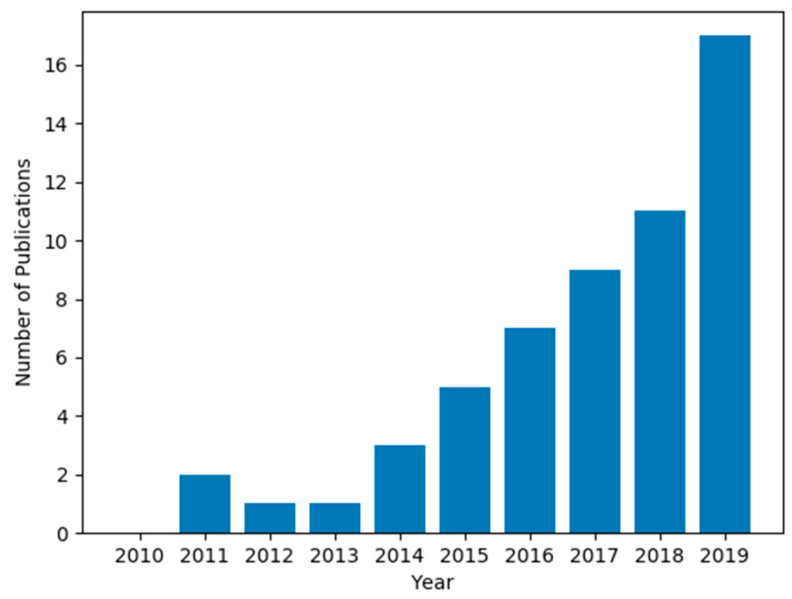
The number of publications examined within the review, segmented by the original publication date up to the last full year. There were 12 publications reviewed between January 2020 and August 2020.

**Figure A2:**
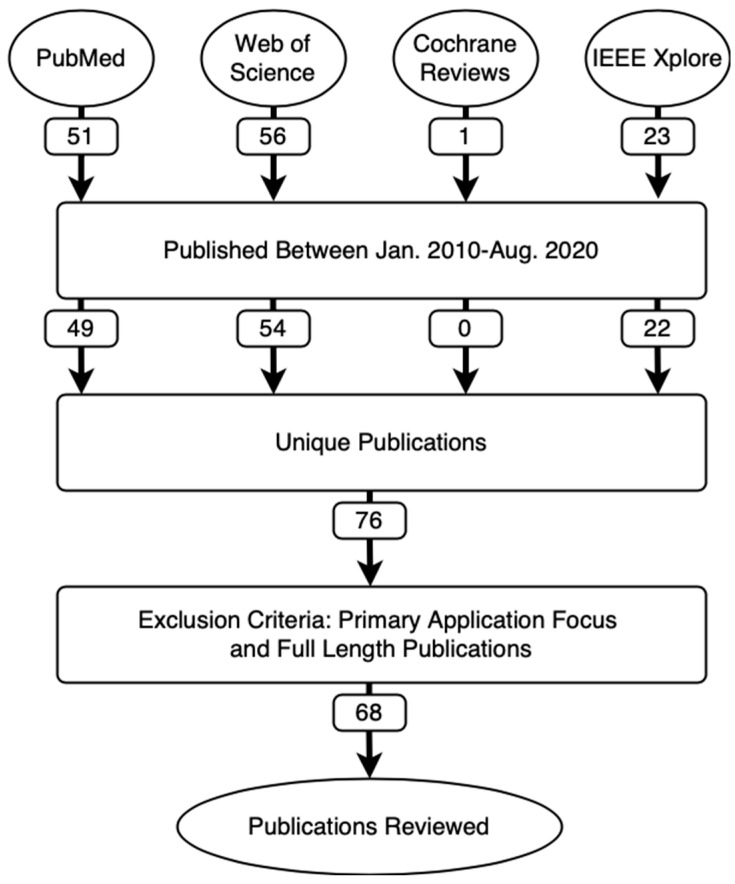
The description of the database keyword search results per database. Each number located within a square indicates the number of returned publications. The exclusion criteria are described in Section 2.

We performed a comprehensive search for studies that applied ML techniques in conjunction with DBS treatment of PD. This search encompassed all relevant journal, review, and conference publications cataloged on four common computerized database search engines: (1) PubMed, (2) the Institute for Scientific Information’s Web of Science, (3) the Cochrane Database of Systematic Reviews, and (4) IEEE’s Xplore Digital Library. We utilized the computerized databases’ keyword search functions using a combination of three key phrases: (1) “Parkinson’s Disease,” (2) “Deep Brain Stimulation,” and (3) “Machine Learning.” We limited our results to publications that were published between January 2010 and August 2020. We further limited our results to English language manuscripts. This initial search identified 76 potential publications. These were evaluated with the predetermined exclusion criteria: (1) PD was not the primary application focus (seven papers were excluded), and (2) papers that contained only published abstracts (one paper was excluded). This process returned 68 publications that were included in this review. The number of publications per year and the number of papers per database are provided in Figure A1 and Figure A2, respectively, in Appendix B.

## 3. DBS Candidate Selection

A key challenge for clinicians is to properly identify DBS candidates who will receive measurable clinical benefits with minimal surgical and stimulation related risk. A large-scale ML assisted analysis was performed to identify patients who may experience complications from DBS surgery. Specifically, Farrokhi et al. [34] examined a retrospective cohort of 501 DBS patients and determined presurgical features that indicated a greater chance of DBS complications. Logistic regression was applied to evaluate risk factors. The model was able to correctly identify subjects that would have any complications (AUC 0.86) and complications within 12 months (AUC 0.91) following DBS surgery. They found that age body mass index, procedure side, gender, and diagnosis of PD were all prominent features.

The screening of potential DBS patients can be limited through visits conducted virtually. There is a growing body of research that utilizes video recordings and ML to determine symptom scores for use in telemedicine. Das et al. [35] compared DBS patients on and off stimulation and classified severe versus mild symptoms using full-body capture video. This analysis was performed for six DBS subjects and classified through SVM. Yohanandan and colleagues [36] used wearable sensors with RF models and predicted tremor severity following the Bain–Findley tremor rating scale (BTRS) with 81% accuracy for use in telemedicine. These studies not only demonstrate the feasibility of using ML in remote monitoring platforms, but also provide early insights into developing a way to classify symptom severity or patients’ phenotypes virtually.

### 3.1. Predictive Motor Biomarkers

Several studies have deciphered motor classifiers by comparing certain motor response differences between healthy and Parkinson’s subjects [37,38,39,40]. Kuhner et al. [37,38] used RF algorithms to stratify 14 PD patients with (DBS-ON) or without (DBS-OFF) stimulation from 26 healthy subjects using a variety of motor control tasks. They found a 94.6% accuracy in distinguishing DBS-OFF patients from healthy subjects when using a standing up test. Another study [38] performed 3D motion capture and gait analysis on 26 PD patients (seven with STN-DBS) and 25 healthy subjects. They identified classifiers (a combination of low-order time derivatives) that performed best at distinguishing DBS-OFF patients from healthy subjects. Such classifying features could then be applied to a large-scale study of gait analysis. Similarly, Huo et al. [39] developed a unique multi-sensor motor response test and utilized KNN to classify 33 subjects as either having PD or being healthy with a 96.6% accuracy.

### 3.2. Non-Motor Considerations in Candidate Selection

Non-motor symptoms (NMS) of PD include neurobehavioral dysfunction, dysautonomia, and sleep dysfunction. NMS treatment response has varied with DBS [41,42,43], with several of these symptoms (i.e., sleep, pain, impulse control disorders, anxiety, and nonmotor fluctuations) showing some degree of improvement. However, with the limited number of studies investigating NMS changes with DBS, the mechanisms underlying the responses are postulated to be related to direct stimulation effects on the anatomic substrate (i.e., STN), postoperative dopaminergic medication reductions, or indirect relation to motoric benefit. Since NMS impacts the quality of life in PD patients, factoring it into the surgical decision-making process is an important clinical consideration.

Koch et al. [44] studied 40 DBS patients’ preoperative electroencephalograms (EEGs) to identify those who would have postoperative cognitive deterioration using an RF algorithm. The data set was balanced in cognitive performance outcomes. The best performing model used both EEG and clinical features and achieved an accuracy of 91%. Chen et al. [45] examined the sleep cycle characteristics of 12 DBS patients. These patients were classified by SVM and decision trees based on local field potential (LFP) signals from the implanted target (STN). They found that alpha, beta, and gamma brain waves were the important predictors in sleep monitoring. Paliwal et al. [46] explored the effect that DBS had on impulsivity in decision making. In this study, 38 PD patients played a virtual slot machine before and after beginning DBS treatment. They applied a hierarchical Gaussian filter model that determined gambling behaviors. They found that DBS patients had increased volatility in relationship to impulsivity. Further, Chrabaszcz et al. [47] examined the LFP signals of 11 patients with STN-DBS while they read three-phoneme words. They were able to identify tongue or lip articulation, specific to DBS patients, that showed that DBS affected some patients’ speech patterns.

## 4. Programming Optimization

Once potential DBS patients have been identified, the optimization of stimulation oftentimes requires frequent and time-consuming programming sessions conducted over 3–6 months from implantation. The programming strategies, coupled with simultaneous medication adjustments, rely a great deal on clinical trial and error. The efficient optimization of strategic DBS treatment planning using ML has been investigated. For example, Khojandi et al. [48] performed a retrospective analysis on 20 STN-DBS patients’ Unified Parkinson’s Disease Rating Scale (UPDRS) III scores. UPDRS [49] is a standard, subjective clinical measurement of PD symptom severity. Khojandi et al. developed an RF algorithm based on preoperative patient and disease characteristics to distinguish patients’ optimized stimulation frequency (high and 60 Hz) with 95% accuracy. Such a model could enable more efficient optimization of DBS parameters. Additionally, Shamir et al. [50] predicted the expected stimulation and medication dosage based on three ML methods, specifically SVM, NB, and RF, for ten patients using 89 post-DBS surgical visits. These visits were used to create a clinical decision support system. This system enabled the correct prediction of motor improvement scores one year after surgery at 86% accuracy. The best performing model that classified individual symptoms varied. For example, SVM predicted tremor and speech outcomes with up to 100% and 93% accuracy, respectively, whereas RF outperformed SVM in terms of axial akinetic symptom prediction, with 86% accuracy.

Khodakarami et al. [51] examined the role of wearable sensors, specifically the personal kinetigraph, in an on/off stimulation classification problem. Their linear regression models determined classification with an AUC of 0.92. Baumgarten et al. [52] examined ten patients with GPi implanted DBS for pyramidal tract side effects (PTSE). An NN classified PTSE in patients whose parameters had been designed to trigger PTSE by clinicians. Each PTSE event was identified by professionals. A positive predictive value (PPV) of over 0.8 was achieved in this study.

Due to the clinical significance of the UPDRS, several publications explored the prediction of the UPDRS score as a response variable. Przybyszewski et al. [53,54] used data mining and decision tree approaches to predict UPDRS III scores based on reflexive saccades with 76% accuracy for 41 PD patients (18 with STN-DBS), and consequently using UPRDRS predictions, they estimated the disease progression in a cohort of PD-DBS patients. Candamil et al. [55] successfully categorized four subjects’ UPDRS scores through linear machine learning based on a copy/draw test that could be applied to calibrate stimulation parameters. A study [56] that applied KNN on seven DBS subjects was able to predict UPDRS symptom severity with over 90% accuracy using wearable sensors on the most affected arm. The authors determined the potential benefit for home-based objective sensor measurements for improving DBS parameter optimization.

### Adaptive DBS

Adaptive DBS (aDBS) refers to automated stimulation based on the real-time feedback of electrophysiology or wearable sensor signals [8,57]. Validated input signals, including basal ganglia local field potentials and especially beta band activity [8,58,59,60], are deemed to be associated with rigidity and bradykinesia (further review of electrophysiology-based aDBS is provided in [61]). Alternative input signals rely on wearable sensors [55,56,62,63,64] that can measure tremor, bradykinesia, dyskinesia, and gait [62]. The fidelity of tremor detection in calibrating a closed loop adaptive system has been demonstrated in several studies [65,66]. The use of ML in tremor classification for DBS patients was also explored and deemed as a useful tool for adaptive, patient-specific optimization [49,62,63,67,68,69,70,71]. Khobragade et al. [62] examined the surface electromyography of two patients (one PD-DBS) over two sessions and analyzed the signals using an NN. They achieved over 80% accuracy when detecting tremor using an NN trained on the same session. However, there was a nearly 50% decrease in performance for repeat patient measurement taken a week apart, which indicated the high variability of tremor symptoms and the benefits from aDBS, which is capable of real-time performance. To that point, LeMoyne et al. [67] surveyed the use of the BioStamp nPoint, a data acquisition device for tremor detection, specifically with the use of an NN, to identify stimulation parameters based on tremor response for future use in aDBS. Camara et al. [63] examined four STN DBS patients and classified tremor based on LFP signals. The authors suggested that a simple threshold would be insufficient to classify tremors. Therefore, they instead used ε-recurrence networks to classify tremors, and consequently applied an SVM to adaptively start or stop stimulation. This process averaged over 90% accuracy at stopping stimulation and over 78% accuracy at starting the stimulation. Shah et al. [49] used a logistic regression algorithm to classify tremor and non-tremor periods in seven DBS subjects using wearable sensor (accelerometer) data. The data set was labeled based on the frequency and duration of accelerometer events. The model achieved an AUC of 0.78 ± 0.11 for the identification of tremor periods. Additionally, Yao et al. [68,69] showcased the benefits of Kalman filtering when applied to tremor classification using multiple ML methods (SVM, RF, KNN, etc.), with as high as 11% classification improvement.

Additionally, ML has been applied to freezing of gait (FoG) [64], boundary decisions [72,73], and PD stages [74] for use in aDBS. However, the clinical feasibility of detecting these non-tremor motor symptoms have yet to be clinically validated in such a system.

Classifying human behavior from brain signals has also been explored in developing closed loop aDBS [75,76,77,78]. The real-time classification of human behaviors would allow for adaptively optimizing stimulation parameters per task. Golshan et al. [50] used variants of SVM classifiers to predict a variety of human behaviors, including speech, mouth movements, arm movements, finger movements, and a button press, with 70% accuracy, and demonstrated the effects of medication on STN-LFP [77]. Mamun et al. [79] decoded movement behaviors from LFPs of the STN and GPi from 12 PD and dystonia subjects with DBS by conducting a finger-clicking task with an accuracy of 99.8%. Furthermore, Niketeghad et al. [80] used SVM from 14 patients’ LFPs to recognize speech, motor, and random type tasks with 73.2% accuracy. Additionally, several other studies examined the classification of behavioral actions using hidden Markov models (HMMs) in combination with the ML techniques [81,82,83]. For example, Jiang et al [83] created an HMM in combination with ML for the classification of motor, language, and motor/language combination tasks. They examined the LFP signals of nine DBS patients using their HMM model, and achieved over 80% accuracy at distinguishing between a combination of any two tasks.

## 5. Surgical Targeting

The surgical implantation of DBS electrodes necessitates accurate real-time identification of a specific brain region. Image guidance with direct magnetic resonance imaging (MRI) based targeting or co-registration of computed tomography (CT) with a 3-Tesla MRI are currently used to localize anatomic targets (i.e, STN and GPi) [28,84,85]. The selection of targets and surgical approaches varies among DBS centers, with microelectrode recording (MER) and intraoperative stimulation effects used to refine electrode placement [86]. ML techniques have been proposed to utilize microelectrode recordings to aid in subthalamic nucleus classification and thereby facilitate electrode placement. Cardona et al. [87,88] performed a retrospective analysis of MER region localization of 10 patients. They compared traditional ML classifiers (NB, SVM, KNN, etc.) with a multi-output Gaussian approach. The relative performance of the classifiers varied by task, but each achieved an accuracy of over 70%. The classification of other specific brain regions through MER signals has also been explored. For example, Khosravi et al. [89,90,91] analyzed the MERs of electrode trajectories with ML techniques over several studies. They retrospectively analyzed 100 DBS subjects’ physicians determinations of optimal implantation trajectory’s MERs with a deep NN and obtained an accuracy of 92% in localizing specific brain regions. Additionally, they utilized unsupervised machine learning techniques on 50 subjects’ MERs and determined the dorsal border of the STN region with 80% accuracy. Finally, a fast Fourier transform was applied to the MERs of 20 DBS surgeries, and a logistic regression algorithm and SVM were applied to localize STN depth. This achieved an accuracy of 85% in detecting the borders of STN. High accuracy region SVM classification models were used by Guillén et al. [92,93] with a 99.5% accuracy at stratifying MERs between the STN, thalamus nucleus, and the substantia nigra. Similarly, Lu et al. [94] applied SVM to 16 subjects’ MERs guided by 3-Tesla MRIs. This model achieved an AUC of 0.87. It is important to consider that MER measurements can be confounded based on the patient’s state of sedation: minimal (awake), light, or deep (general anesthesia).

Alternative approaches, such as the utilization of traditional MRIs [50] and state-of-the-art 7-Tesla MRIs [95,96] or magnetoencephalography (MEG) [97] in conjunction with ML techniques, have been rarely explored. Ozturk et al. [98] examined ten patients’ LFP signals and determined STN localization by an online linear discriminant classifier. Similarly, Darbin et al. [99] used LFP signals in a Tree Bagger algorithm to identify regions in non-human primates. Stuart et al. [100] examined 16 subjects’ EEGs with multiple stimulation regions specifically: GPI, VIM, or STN. The fast Fourier transform was used to extract features. They compared the performance of several ML classifiers in determining the stimulation region. A decision tree based gradient booster paired with principal component analysis (PCA) performed the best at region identification (F1 0.62, precision 0.68).

ML has also been used in conjunction with HMMs to model surgical planning. For example, Valsky et al. [101] developed a real-time ML classification of the subthalamic border for accurate detection of the STN in DBS surgery. They examined 58 STN trajectories using an SVM and achieved an accuracy of 97.6%. They used the SVM results to inform an HMM for real-time identification, and this model was tested on an additional 73 trajectories, achieving an accuracy of 94%. Valsky et al. then continued this work in [102] for the identification of the striatopallidal border site to the GPi. They applied a similar SVM/HMM algorithm to 116 trajectories over three patient types: awake PD patients, lightly anesthetized genetic, and non-genetic dystonia subjects. Their algorithm had similar performance to clinical experts at GPi border identification across all patient types considered.

## 6. Insights into DBS Mechanisms

A small body of work exists that aims to better understand the underlying biological and chemical signals that impact DBS performance. Trevathan and colleagues [103] used NN and Volterra kernels to characterize stimulation-evoked neurochemical releases. They compared their proposed frameworks of stimulation-evoked dopamine releases in several animal models. This allowed for a neurochemical signaling comparison of healthy and diseased brains, which theoretically could be used to develop strategies for controlling DBS systems. In another study, Khawaldeh et al. [104] examined the off medication LFPs of 18 STN-DBS patients. They used NB classifiers for movement predictions and determined the band frequency of oscillatory synchronization associated with motor impairment in PD.

Finally, ML techniques have been utilized in the design of DBS systems. Jovanov et al. [105] developed a unique aDBS system hardware platform that allowed for the real-time optimization of DBS treatments by utilizing a genetic algorithm to identify patterns in treatment planning. Other studies that concentrate on hardware limitations include: Zhu et al. [106], which focused on performance, memory, and hardware requirements for seizure detection tasks in PD using EEG/ECoG (electrocorticography) as inputs to a custom decision tree algorithm; and De La Pava et al. [107], which applied hierarchical KNN to reduce the computational load of tissue-activated visualization in DBS modeling.

## 7. Discussion

The application of ML in DBS treatment for PD is a rapidly growing, interdisciplinary field that has unprecedented potential to transform all aspects of DBS. If considered and executed appropriately, ML will prove a tremendously beneficial tool for clinicians. This review examined the role of ML in DBS in the broadest terms, performing an exhaustive review and considering nearly a decade of publications. We found compelling applications for ML in (1) DBS candidate selection; (2) programming optimization; (3) surgical targeting; and (4) insights into DBS mechanisms. Even within a specific category, there was large heterogeneity among the studies regarding feature type, ML models, and practicality of implementation. Broadly speaking, the clinical features utilized in ML studies benefited from objectiveness. As such, ML research favors LFP, wearable sensors, MERs, and similar unbiased measurements.

The identification of PD patients whose motor control symptoms would be more effectively managed under DBS than medication-based therapies is a clear target for ML. Predicting which patients may have adverse effects from DBS therapies is crucial to ensuring optimal patient outcomes. Non-motor symptoms present further complexity to the PD phenotype, which has implications on disease progression, response to therapy (medication and stimulation), and surgical risk. Recent publications have offered methods to predict neurobehavioral changes preoperatively, hence possibly influencing the DBS decision-making process. Measuring motor and non-motor symptomatology through objective means will permit the expansion and robustness of these ML-based decision models. Additionally, patient phenotyping through unsupervised methods may reveal trends in patient data that have not been incorporated into current predictions. The automatic classification of potential DBS patients could efficiently determine an optimized treatment plan, thus dramatically improving patients’ quality of life.

Adaptive DBS has a significant scope of clinical impact on a patient’s quality of life. The use of LFPs and/or wearable sensors serve as robust measurement instruments for the classification of specific behavioral tasks, severity, and/or tremor. However, a large portion of these studies utilized assessments obtained in a clinical setting. Further work is needed to identify motor control symptoms in real-world environments. Tremor detection using wearable sensors appears to be one of the nearest methods to application in the clinical field. Additionally, the development of HMMs that accurately describe a DBS would have a great impact on strategic treatment planning. To that end, dynamic treatment planning would require a further assessment as to the practicality of adaptively tuning DBS parameters.

Surgical targeting utilizing ML to interpret intraoperative signals can be used to enhance the targeting of anatomic substrates, providing immediate clinical relevance as a support tool to assist in surgical planning. However, while many of the studies reviewed and utilized MER signals in classifying or delineating surgical targets to enhance optimal electrode placement, it is important to note that few studies have robustly examined clinically relevant outcomes related to these approaches. Image-based training of surrounding anatomy in ML modeling to enhance surgical target localization is expanding and showing greater precision and adaptability than current direct (image guided) or indirect (Talaraich coordinates) methods [108,109].

Although ML’s application in DBS is relatively early in its development, the preliminary evidence is remarkably promising. Its ability to determine underlying patterns within patient data offers the potential to enhance candidate selection, facilitate surgical targeting, and more efficiently optimize programming approaches to yield rapid and robust clinical outcomes. In general, ML models that have interpretable data structures (explainable AI) should be favored in future studies. This allows experts to translate ML decision-making criteria, and could potentially provide insights into the fundamental nature of DBS. Additional insights may be found in the growing body of literature that proposes using PD-related genetic mutations and genetic profiles to identify DBS therapy effectiveness [110,111]. Furthermore, ML methods generally benefit from large data sets. The majority of studies considered in this review are limited by their small number of patients. Before any of the presented methods can be applied in a clinical setting, large-scale, randomized, multi-center studies are necessary to ensure an expansive data set while minimizing differences in target selection and surgical planning.

## Appendix A

Several statistical metrics are frequently calculated to measure model performance in classification tasks. These include:

- Precision: the measurement of positive and negative results that are truly positive
- Recall: the proportion of positives that are correctly identified
- Accuracy: the measurement of correct predictions out of all predictions
- PPV: the proportion of positive predictions that are true positives
- F1 Score: the harmonic average of precision and recall
- AUC under the receiver operating characteristic: the aggregate comparison of the true positive rate and the false positive rate at different classification thresholds, providing an overall performance metric

Similarly, several statistical metrics are frequently used to measure model performance in regressions tasks. These include:

- MSE: the average squared difference between the predicted and actual values
- MAE: the average of absolute errors between the predicted and actual values
- MAPE: the average percentage error between the predicted and actual values

## Appendix B

After exclusion criteria were applied, we reviewed 68 publications from four databases. The number of publications that explore machine learning’s role in deep brain stimulation as a treatment of Parkinson’s disease is rapidly expanding. Figure A1 shows the number of publications within our review, segmented by publication year.
